# Druggable Protein Interaction Sites Are More Predisposed to Surface Pocket Formation than the Rest of the Protein Surface

**DOI:** 10.1371/journal.pcbi.1002951

**Published:** 2013-03-07

**Authors:** David K. Johnson, John Karanicolas

**Affiliations:** 1 Center for Bioinformatics, University of Kansas, Lawrence, Kansas, United States of America; 2 Department of Molecular Biosciences, University of Kansas, Lawrence, Kansas, United States of America; University of Houston, United States of America

## Abstract

Despite intense interest and considerable effort via high-throughput screening, there are few examples of small molecules that directly inhibit protein-protein interactions. This suggests that many protein interaction surfaces may not be intrinsically “druggable” by small molecules, and elevates in importance the few successful examples as model systems for improving our fundamental understanding of druggability. Here we describe an approach for exploring protein fluctuations enriched in conformations containing surface pockets suitable for small molecule binding. Starting from a set of seven unbound protein structures, we find that the presence of low-energy pocket-containing conformations is indeed a signature of druggable protein interaction sites and that analogous surface pockets are not formed elsewhere on the protein. We further find that ensembles of conformations generated with this biased approach structurally resemble known inhibitor-bound structures more closely than equivalent ensembles of unbiased conformations. Collectively these results suggest that “druggability” is a property encoded on a protein surface through its propensity to form pockets, and inspire a model in which the crude features of the predisposed pocket(s) restrict the range of complementary ligands; additional smaller conformational changes then respond to details of a particular ligand. We anticipate that the insights described here will prove useful in selecting protein targets for therapeutic intervention.

## Author Summary

Identifying small-molecule inhibitors of protein interactions has traditionally presented a challenge for modern screening methods, despite interest stemming from the fact that such interactions comprise the underlying mechanisms for cell proliferation, differentiation, and survival. This suggests that many protein interaction surfaces may not be intrinsically “druggable” by small molecules, and elevates in importance the few successful examples as model systems for improving our understanding of factors contributing to druggability. Here we describe a new approach for exploring protein fluctuations leading to surface pockets suitable for small molecule binding. We find that the presence of such pockets is indeed a signature of druggable protein interaction sites, suggesting that “druggability” is a property encoded on a protein surface through its propensity to form pockets. We anticipate that the insights described here will prove useful in selecting protein targets for therapeutic intervention.

## Introduction

Manipulating the interactions between proteins represents a promising avenue for therapeutic intervention in a variety of settings. Given the ubiquitous nature of protein interactions, selectively manipulating such interactions could serve as a means to treat conditions including viral and bacterial infections, cancer, and autoimmune disorders [1]–[7]. In spite of recent ongoing efforts that have provided cause for optimism, protein interactions continue to be viewed as a challenging class of therapeutic target [8]–[12]. While high-throughput screening efforts that fail to yield extensive hits are typically not reported in the literature, hit rates as low as 0.01% in a large pharmaceutical library have been described [13].

This dearth of successful representatives to study has given increased importance to the several cases in which a protein structure has been solved in complex with a biological protein partner and also in complex with a small molecule inhibitor. Wells and McClendon [8] compared six such cases and observed that binding was not associated with a large conformational change in any of these examples; and yet, the concave pocket on the protein surface at which the small molecule binds was typically smaller or not present in the unbound protein structure. In order for inhibitor binding to occur, the surface of the unbound structure therefore had to undergo local rearrangement to reveal a small molecule binding site that would not necessarily be evident from the unbound structure [8].

Given the limited success in identifying modulators of protein-protein interactions, it has proven helpful at an early stage to validate a protein surface site by evaluating its “druggability”. As such, fragment-based methods have been developed to experimentally assess the druggability of a protein interaction site by determining which members of a small molecule probe set bind to a target protein, and where on the protein surface these bind. This experiment can be conducted using “SAR by NMR” [14], which tracks chemical shift differences to identify binding sites on the protein surface, or by the “multiple solvent crystal structures” method [15], in which independent structures of the target protein are solved after soaking with a collection of organic solvents. Both of these approaches aim to probe the regions of a protein surface that can accommodate small-molecule binding, with a preference for sites that are not uniquely disposed to bind a particular pre-selected ligand. In either technique, specific regions of the protein surface that interact with a variety of probe molecules – albeit weakly – are inferred to be a putative site for more potent binding by some yet unidentified compound.

Inspired by these methods, we hypothesize that the ability to form a binding pocket may be the limiting factor for druggability of a protein surface site. We further propose that compounds identified in biochemical screens as inhibitors of protein interactions result from natural shape complementarity to specific surface pockets that form with little energetic cost to the protein. Together these hypotheses imply that druggable sites differ from the rest of the protein surface, in that fluctuations under physiological conditions at druggable sites include a special subset of “pocket-containing” conformations.

To test these hypotheses, we have developed computational methodology to explore protein fluctuations in a biased way, by providing a driving force towards conformations in which a surface pocket is present. Several other studies have generated ensembles of protein conformations reflecting fluctuations around the native state and used these either to assess druggability [16] or as a starting point for docking studies [17]–[24]. Because these ensembles are generated in an unbiased manner, however, a large fraction of the resulting ensembles correspond to protein conformations that are dissimilar to the corresponding bound structure in both pocket size and hydrophobicity [18]. One of these studies found that carrying out simulations in methanol led to formation of surface pockets which could accommodate small molecules [25], but the use of this non-biological solvent may lead to unphysical artifacts in the resulting models. Molecular dynamics has also been used in a computational analog of “SAR by NMR” in which simulations are carried out using an explicit mixed solvent, allowing druggable sites to be identified by locating accumulation of probe molecules [17], [20], [26]. Though these methods proved effective for binding sites, the unbiased nature of the underlying simulations make these approaches very computationally intensive. Recently Kozakov et al. have developed a computational analog of the multiple solvent crystal structures method, by using docking to identify “consensus” sites at which several probe molecules cluster [27]. They confirmed that these molecular probes indeed cluster at established druggable sites and that known inhibitors often occupy these consensus sites. Such an approach, however, cannot efficiently explore surface pockets that form via concerted motions involving the protein backbone due to the computational expense associated with repeatedly docking multiple small molecule probes. The biasing potential we describe here avoids this limitation by not needing to dock probe molecules, and therefore can be used in the course of a simulation that samples a broader range of conformational fluctuations.

## Results

### Quantitative analysis of surface pockets

Because a wide, shallow pocket that is unsuitable for small molecule binding can have the same volume as a deep pocket that is more suitable for small molecule binding, we introduce the concept of “deep” volume of a pocket: the volume of the pocket that is well-sequestered away from solvent. To quantitatively identify small molecule binding pockets and measure their “deep” volume, we implemented a modified version of the LIGSITE^cs^ algorithm [28]. This approach starts by creating a grid around a protein and marking each grid point as occupied by protein, surface, or solvent. Next, the algorithm performs linear searches on the grid to find “Surface-Solvent-Surface” events: lines drawn between two surface points that pass through only solvent (**Figure S1**), indicating a concave region on the protein surface. To distinguish between total pocket volume and deep volume, pocket points that fall within 2.5 Å of solvent are marked as “surface pocket” points and are excluded from the “deep volume” calculation. Finally, the remaining contiguous points involved in these events are clustered into “deep pockets”. As expected, the deep pocket volumes we use here are correlated to, but smaller than, pocket volumes found by other pocket detection methods, such as Q-SiteFinder [19] (**Figure S2**). Our implementation differs from the original LIGSITE^cs^ algorithm [28] in that our search is restricted to the region around a specific “target” residue on the protein surface, allowing us to rapidly test for pockets at a specific surface site. Additional minor differences are described in **Text S1**.

**Figure 1:**
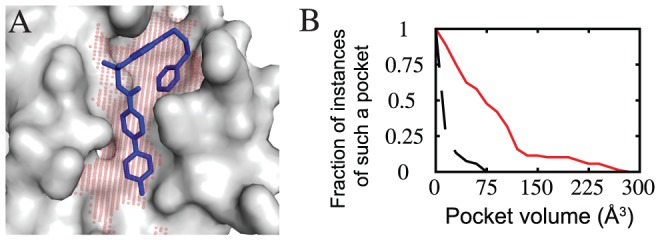
Identifying surface binding pockets. (**A**) Bcl-X_L_ (*grey surface*) is shown in complex with an inhibitor (*blue sticks*). The protein surface features a large pocket (*red spheres*) complementary in shape to the inhibitor. (**B**) Deep pocket volumes of surface pockets at protein interaction sites harboring a bound inhibitor (*red line*) are larger than those found elsewhere on the protein surface (*black line*). Data are collected from a test set of seven proteins, each of which has been solved in complex with a small-molecule inhibitor (Bcl-X_L_, IL-2, FKBP12, HPV E2, ZipA, MDM2, and the BIR3 domain of XIAP).

A demonstration of this method is shown in **Figure 1A**. Bcl-X_L_ is an anti-apoptotic protein in the Bcl-2 family whose over-expression has been implicated in the survival of cancer cells. A series of acyl-sulfonamide-based ligands have been shown to inhibit Bcl-X_L_ activity by competing for its peptide-binding groove. Here, we have removed one such inhibitor from a co-crystal structure and applied our modified implementation of the LIGSITE^cs^ algorithm at this surface site. The resulting pocket has intuitive shape complementarity to the ligand even though it was generated from the protein structure without the ligand present. This is unsurprising, given that the ligand occupying this pocket is complementary in shape to the protein surface.

We compiled a test set of all seven proteins for which structures have been solved both alone and in complex with a small-molecule inhibitor bound to a protein interaction site (Bcl-X_L_, IL-2, FKBP12, HPV E2, ZipA, MDM2, and the BIR3 domain of XIAP) (**Table 1**). We compared the deep pocket volume at randomly selected regions of the protein surface (see **Text S1**) to the deep volume of the inhibitor-bound pocket. Since the pocket definition can depend somewhat on the particular “target” residue used, the deep volume of the inhibitor-bound pocket was measured several times using different target residues. As shown in **Figure 1B**, across all seven proteins, most pockets identified by this algorithm at randomly selected sites (*black line*) on the protein surface have a deep volume smaller than 25 Å^3^, and all are smaller than 75 Å^3^. In contrast, the distribution of inhibitor-bound pocket volumes (*red line*) is significantly shifted, with about half of the inhibitor-bound deep pocket volumes larger than 75 Å^3^. This observation is consistent with results generated using other pocket detection methods [29]–[33], although those other studies were not focused on inhibitors of protein-protein interactions.

**Table 1:** Our test set is comprised of proteins for which structures have been solved both alone and in complex with a small-molecule inhibitor bound to the protein interaction site.

Protein	Partner	Number of residues	Number of residues used in simulations	Deep pocket volume from unbound protein structure (Å^3^)	Deep pocket volume from protein-protein complex (Å^3^)	Deep pocket volume from inhibitor-bound complex (Å^3^)	Deep pocket volume from conformations generated via biased simulations (the 5% with lowest energy) (Å^3^)	Affinity of most potent known inhibitor (nM)
MDM2	p53	492	119	129	150	201	194	80
ZipA	FtsZ	328	139	91	166	61	92	12,000
Bcl-xL	Bak	233	141	200	377	342	210	0.5
XIAP	SMAC	497	117	158	27	40	323	67
IL-2	IL-2R	153	128	38	38	38	177	60
HPV E2	HPV E1	367	193	93	92	132	129	40
FKBP12	Tbeta1-R	108	107	90	124	145	116	7

### Druggability of protein surface sites

The results presented above demonstrate that inhibitor binding occurs at surface sites containing a pocket, and that these sites are distinct from the remainder of the protein surface. We therefore formulated the hypothesis that the ability of the protein surface to form such pockets may be the limiting determinant of the inherent druggability at this site.

Unlike previous standalone methods for pocket detection, we instead implemented our algorithm as a term in the Rosetta [34] energy function alongside the canonical energetic determinants of protein structure such as packing, hydrogen bonding, and solvation (see Methods section). By including this biasing term in the energy function, we may use any of the standard functionalities provided in Rosetta; inclusion of this term, meanwhile, will lead to simultaneous optimization of both “pocket score” *and* the traditional energy terms. In essence, the contribution from the “pocket” term serves as a proxy for the energy associated with binding of some (unspecified) small molecule partner.

**Figure 2:**
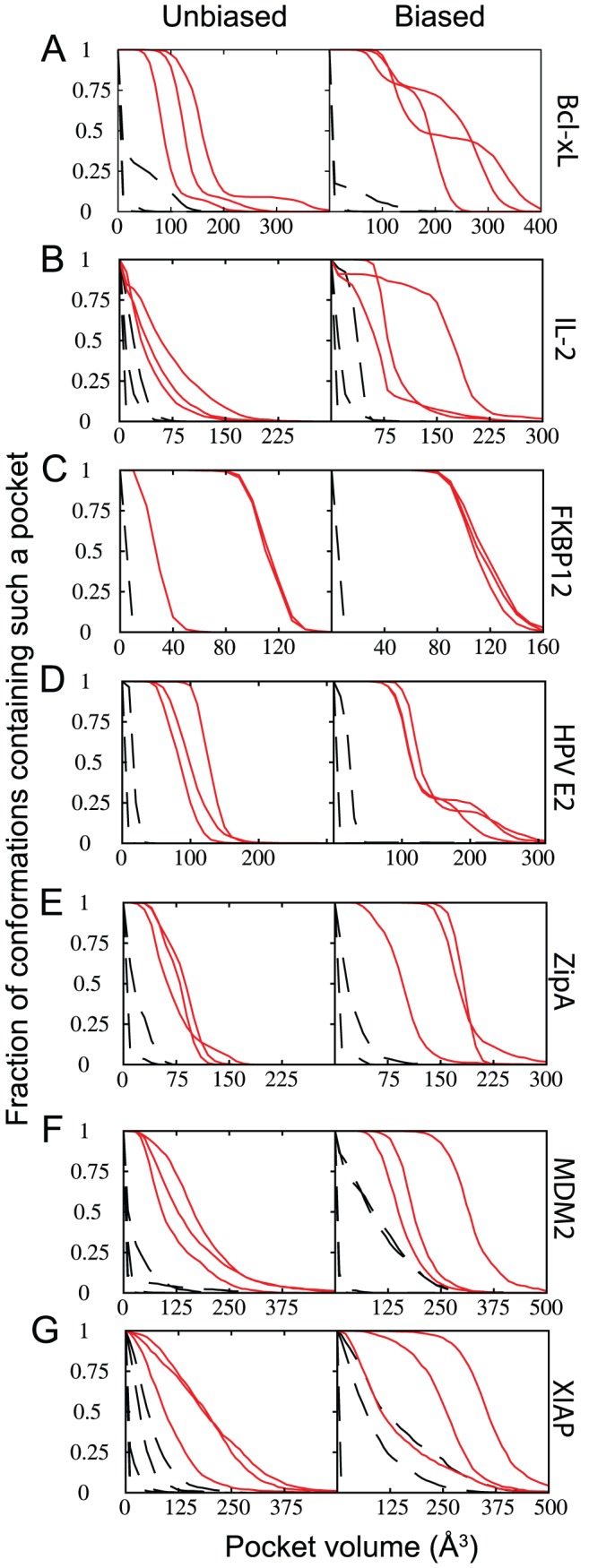
Surface pockets emerge only at druggable sites. Volumes of surface pockets are shown from conformations generated with no biasing potential (*left*) and upon inclusion of a “pocket opening” biasing potential (*right*) for each of the seven proteins that comprise our test set. Surface pockets occur at druggable protein interaction sites (*solid red lines*) more frequently than elsewhere on the protein surface (*dashed black lines*). (**A**) Bcl-X_L_. (**B**) IL-2. (**C**) FKBP12. (**D**) HPV E2. (**E**) ZipA. (**F**) MDM2. (**G**) BIR3 domain of XIAP.

To test the hypothesis that pocket formation may be the limiting determinant of druggability, we performed biased and unbiased simulations on the unbound conformations of Bcl-X_L_, targeting residues at the protein interaction site as well as at equivalent randomly selected residues elsewhere on the protein surface. Surface sites included in the random set were matched to those at the protein interaction site on the basis of their secondary structure, and further that a contacting pocket of equivalent size to that of the protein interaction site (evaluated by Q-SiteFinder [19]) was present in the unbound conformation (see **Text S1** and **Figure S3**); further, the random sites were each at least 12 Å from one another. Both backbone and sidechain degrees of freedom were allowed to move during simulations (see Methods section). The deep pocket volumes from each of 1,000 conformations generated via each method are shown as cumulative histograms in **Figure 2A**. Pockets at the protein interaction site (*solid red lines*) form more often and are significantly larger than those formed elsewhere on the protein surface (*dashed black lines*). The largest pockets in the biased simulations are sampled with much higher frequency than in the corresponding unbiased simulations (**Figure 2A**), demonstrating that the biasing potential drives sampling towards these conformations. These observations further hold for each of the other six additional proteins comprising our test set (**Figures 2B–G**), and also after inclusion of additional random sites (**Figure S4**) or starting from the protein-bound conformation (**Figure S5**).

**Figure 3:**
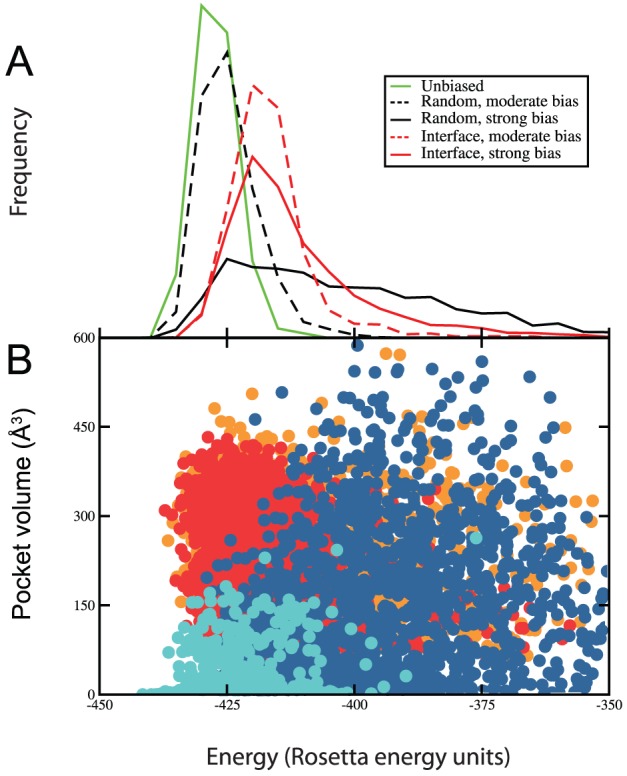
Energetic analysis of Bcl-X_L_ pocket opening. (**A**) Conformations generated without the use of a biasing potential (*solid green line*) show a similar distribution of energies to those generated with the biasing potential at a randomly selected target residue (*dashed black line*); increasing the strength of the biasing potential here leads to conformations with higher energies (*solid black line*). In contrast, application of the biasing potential at the protein interaction site (*red lines*) leads to conformations with a distribution of energies that strongly overlaps with those energies of conformations sampled in the unbiased simulations, suggesting that these conformations represent low-energy states accessible to the unbound protein. (**B**) A scatterplot showing the deep pocket volume for conformations generated with the biasing potential applied to one of the random sites (*moderate bias in cyan, strong bias in blue*) or to the protein interaction site (*moderate bias in red, strong bias in orange*). Low-energy conformations containing large pockets are sampled only if the biasing potential it is applied at the protein interaction site; while large pockets are sampled using the strong bias at random sites, these conformations have considerably higher energy. All energies shown here were evaluated in the absence of the biasing potential, for fair comparison.

To examine the physiological relevance of the conformations generated in biased simulations, we compared their energies to those obtained in equivalent unbiased simulations. For Bcl-X_L_, we used Rosetta to evaluate the (unbiased) energy for each of 1000 conformations generated from an unbiased simulation, a simulation with the biasing term applied to either the protein interaction site or a random surface residue, and equivalent simulations in which the weight of the biasing term was increased tenfold. In all cases we evaluated energies *without* contribution from the biasing term; a histogram of these energies is shown in **Figure 3A**. Conformations from the unbiased simulation (*green solid line*) have a very similar distribution of energies as conformations from a simulation in which the biasing potential was applied to a randomly selected target residue (*black dashed line*); this is unsurprising given that few of these conformations contain pockets, implying that a similar ensemble of conformations are sampled. Applying a stronger weight to the biasing potential using the same randomly selected target residue (*solid black line*) leads to conformations that are far less energetically favorable, indicating that pocket opening to satisfy the biasing potential could not be achieved without extensive energetic cost to the protein. In contrast, applying the biasing potential to a residue at the protein interaction site led to conformations that were only slightly higher in energy (and with overlapping distributions) than those conformations sampled in the unbiased simulation, for either weight of the biasing potential (*red lines*). A scatterplot showing the deep pocket volume for each of these conformations highlights the fact that conformations containing large pockets are sampled with the moderate biasing potential *only* if it is applied at the protein interaction site (**Figure 3B**, *cyan vs. red points*). Application of the stronger biasing potential to random (carefully matched) surface sites leads to generations of low-energy conformations without large pockets, and also pocket-containing conformations with much higher energy (**Figure 3B**, *blue points*).

The same observations also hold for each of the other six proteins comprising our test set (**Figure S6**, **Table 1**). Collectively, these results demonstrate that pocket opening at the druggable site can occur with little energetic cost to the protein, while pocket opening elsewhere on the protein surface requires that the protein adopt a highly unfavorable conformation.

It is notable that in each of these seven examples surface pockets were identified at the protein interaction site. It is equally notable, however, that similar surface pockets were *not* observed elsewhere on the protein surface (**Figure 2**). This comparison highlights the qualitative difference between the protein interaction site – already demonstrated to be druggable in a practical sense for each of these examples – and the remainder of the protein surface, at which high-affinity interactions with small molecules have not been observed.

### Pocket shapes are encoded on the protein surface

The results presented above demonstrate that there is a natural predisposition towards pocket formation in certain druggable regions on the protein surface. We next asked whether these preferred pocket-containing conformations dictate the range of potential small-molecule inhibitors suitable for binding at this site. Should this be the case, protein conformations generated using a biasing potential to induce pocket formation should resemble inhibitor-bound conformations more than conformations generated using an equivalent unbiased protocol.

**Figure 4:**
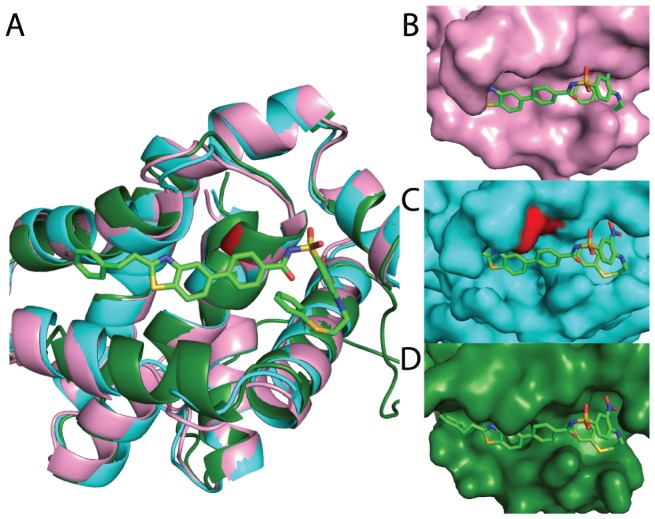
Representative conformations of Bcl-X_L_ . An unbound crystal structure (*pink*), an inhibitor-bound crystal structure (*green, with inhibitor shown in sticks*), and a low-energy conformation generated from the unbound crystal structure using the biasing potential (*cyan, with target residue in red*) are shown. (**A**) The overall protein architecture is preserved amongst all three; movement of the helix in the foreground upon binding is not recapitulated in the pocket-opened conformation. (**B–D**) The pocket revealed in this low-energy conformation nonetheless strongly resembles the surface pocket in the bound crystal structure, and even bears shape-complementarity to the inhibitor. The identity of the inhibitor was not used in generating this conformation, but was added retrospectively for visual comparison.

Turning first to Bcl-X_L_, we aligned the lowest-energy conformation produced from the biased simulations described earlier (which were started from the unbound protein structure) to both unbound and inhibitor-bound crystal structures (**Figure 4A**). Direct superposition of the inhibitor from the bound structure onto the unbound crystal structure reveals extensive steric clashes (**Figure 4B**), highlighting the local protein conformational changes that must take place in order for inhibitor binding to occur. Remarkably, examination of the conformation from the biased simulation shows that part of the protein surface has adopted a shape highly complementary to the inhibitor (**Figure 4C** **, right side**) – even though no information about the identity of the inhibitor was included in the simulation that produced this conformation. Further, this conformational change occurred without direct involvement of the “target” residue at which the biasing term was applied.

On the other hand, the conformation from the biased simulation did not recapitulate the bound conformation exactly: the latter contains an extension to the surface pocket (left side in the orientation shown) to accommodate the “tail” of the inhibitor (**Figure 4D**). Comparison of the protein backbone in all three structures (**Figure 4A**) reveals the protein structural reorganization required to accommodate this “tail”: the helix below the inhibitor (**Figure 4A** **, foreground**) unwinds on the left side, and tightens on the right side (in the orientation shown). Because the deep pocket volume is larger in the inhibitor-bound crystal structure than in the conformation from the biased simulation, we anticipate that sampling in these simulations was insufficient to fully recapitulate this highly concerted conformational change.

The diversity of conformational changes associated with inhibitor binding in different proteins is highlighted in **Figure S7**. In IL-2, slight rearrangement of a helix (at top of the orientation shown) allows a sidechain rotamer change that both reveals the binding pocket and creates new interactions with inhibitor shown (**Figure S7A**). In contrast FKBP12, HPV E2 and ZipA each contain a pre-formed pocket on the unbound protein surface that strongly complements the inhibitor, indicating that inhibitor binding occurs primarily via a “lock-and-key” mechanism in these cases (**Figure S7B–D**). Expansion of the ZipA pocket observed in the biased simulations (**Figure 2E**) resulted from sidechain rearrangement on the surface to the left side of the binding site (in the orientation shown), without disrupting the pre-ordered portions of the binding site. Nutlin binding to MDM2, on the other hand, requires splaying apart of two surface helices to create the binding pocket: this conformational change is dramatically recapitulated in the lowest-energy individual conformation from the biased simulations (**Figure S7E**). Finally, unlike these previous examples showing modest conformational changes, binding of a Smac-mimetic to the BIR3 domain of XIAP is associated with extensive rearrangement of long surface loops (**Figure S7F**).

To assess whether the protein conformations generated using the biasing potential resemble inhibitor-bound conformations more closely than conformations generated using an equivalent unbiased protocol, we quantitatively compared the ensembles of conformations produced by each method. We note that neither method uses any knowledge of any particular inhibitor when generating an ensemble of conformations. Therefore, rather than report the protein RMSD in reference to one pre-selected bound conformation we instead individually computed the interface RMSD (iRMSD) relative to every available inhibitor-bound crystal structure (see **Text S1**), and took the lowest of this set of iRMSD values to reflect the suitability of this conformation for binding some (known) unspecified inhibitor.

**Figure 5:**
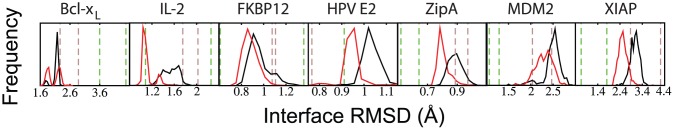
Opening pockets shifts the conformational ensemble towards inhibitor-bound structures. Distributions of RMSD over interface atoms (iRMSD) to the closest inhibitor-bound crystal structure for conformations generated with (*red lines*) or without (*black lines*) the biasing potential. The iRMSDs from the unbound structure to the most and least similar inhibitor-bound crystal structures are also indicated (*dashed brown vertical lines*). For all seven proteins comprising the test set, the biased simulations produced conformations closer to an inhibitor-bound crystal structure than the unbiased simulations.

For each of the seven proteins in our test set described earlier, we show the iRMSD for each member of the biased and unbiased ensembles to its closest inhibitor-bound crystal structure (**Figure 5**). We note that the iRMSD values of conformations sampled vary across these seven different proteins; this stems from the fact discussed earlier that in some cases the unbound starting structure strongly resembles the inhibitor-bound structure (FKBP12, HPV E2, ZipA), while in other cases a more extensive conformational change accompanies binding (MDM2, XIAP) (**Figure S7**). For reference, we also indicate the iRMSD of the unbound starting structure relative to the closest and most distant inhibitor-bound structures (**Figure 5**, *dashed brown lines*) and the protein-bound structure relative to the closest and most distal inhibitor-bound structures (**Figure 5**, *dashed green lines*).

Though there is overlap between the resulting distributions, for every one of the seven proteins we examined the conformations sampled in the biased simulations are closer to an inhibitor-bound conformation than the conformations sampled in the corresponding unbiased simulations. This observation applies not just to the iRMSD of atoms directly involved in binding (**Figure 5**), but to the backbone atoms of the corresponding residues as well (**Figure S8**). Further, in five of the seven cases (Bcl-X_L_, IL-2, FKBP12, ZipA, and XIAP) the conformations generated from the biased simulations are closer than the starting unbound conformation to an inhibitor-bound structure (**Figure 5**). Conformations sampled in simulations of HPV E2 and MDM2 that moved further than the unbound conformation from the inhibitor-bound structures may be due to slight inaccuracies in the Rosetta energy function, or alternatively may be sampling novel truly “druggable” conformations for which complementary inhibitors have not yet been identified.

To further characterize the pockets generated using the biasing potential, we evaluated the percentage of solvent accessible surface area that is hydrophobic (hSASA) for each pocket (**Figure S9**). While the hydrophobicity of the inhibitor-bound pockets vary amongst the different protein test cases, in each case the pockets generated using the biasing potential exhibit similar hydrophobicity to the corresponding inhibitor-bound conformation. Like the shape of these pockets, then, their hydrophobicity appears to be an intrinsic property resulting from details of the surface geometry and composition.

Collectively these results demonstrate that biasing simulations towards conformations in which a surface pocket is present drives the resulting ensemble towards the conformations observed in inhibitor-bound crystal structures. Because no information about the identity of any particular inhibitory compound was included in the biasing potential, this suggests that the general shape (and hydrophobicity) of surface pockets available to a potential inhibitor is an inherent property of the druggable interface itself.

### Druggability of survivin

The examples comprising our test set in the studies above were selected on the basis of a known compound directly inhibiting a protein interaction. For each of these cases, then, the druggable site is coincident with the protein functional site. We next turned to survivin, an example of a protein in which the two sites are non-overlapping.

**Figure 6:**
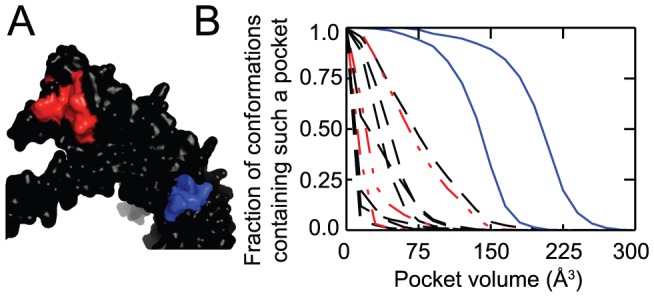
Distinguishing druggable from functional sites on survivin. (**A**) The crystal structure of survivin, showing the protein interaction site (*red*) and the distal druggable site identified by NMR (*blue*) [14]. (**B**) Volumes of surface pockets are compared for conformations generated with the biasing potential applied at random surface residues (*dashed black lines*), applied at the protein interaction site (*dashed red lines*), and applied at the distal druggable site (*solid blue lines*). Pockets emerge at the druggable site but not elsewhere on the protein surface.

Survivin is among the most strongly tumor-specific proteins known [35], is a notable signature of unfavorable disease outcome [36], and has been well-validated as a therapeutic target using an antisense oligonucleotide [37] and a transcriptional repressor [38], [39]. Despite this intense interest, however, no direct inhibitors of survivin function have been identified. Survivin carries dual functions which together explain its important role in cancer: it serves as an inhibitor of apoptosis and is also required for cell division [40]. The anti-apoptotic activity of survivin derives from binding the Smac/DIABLO peptide via a BIR domain. While small-molecule inhibitors of other Smac-binding BIR domains have been identified [41]–[44] (we included the XIAP BIR domain in the test set described in previous sections), efforts in this vein using survivin have not yet proven fruitful [40], [45]. Consistent with a hypothesis that the peptide-binding surface of survivin may be intrinsically undruggable, an SAR by NMR approach used by Hajduk et al. found only a single probe that interacted with this site (of 9,370 probes tested) – a full order of magnitude fewer than the number of interacting probes found using proteins for which potent inhibitors have been identified [14]. Intriguingly, this study revealed a separate distal site (**Figure 6A**) at which 33 probe compounds bound, well within the frequency observed for druggable sites on other proteins but not useful as a starting point for modulating survivin function.

We carried out a series of biased simulations as described earlier, using the unbound crystal structure of survivin as a starting point. In agreement with results from SAR by NMR [14], we find that pockets at the “undruggable” peptide-binding site of the BIR domain do not form with any higher propensity than at randomly selected equivalent surface sites (**Figure 6B**). This result is particularly notable as it stands in contrast to our observations from the XIAP BIR domain, in which surface pockets *are* formed at the peptide-binding site (**Figure 2G**). Though beyond the scope of the current work, we anticipate that further detailed studies may reveal the precise origin of the differing propensity for binding small-molecules exhibited by the peptide-binding groove of these two highly similar BIR domains.

Though large surface pockets do not form at the peptide-binding site of survivin, they *do* form readily at a specific alternate site: the same distal surface identified by Hajduk et al. [14] (**Figure 6B**, *blue lines*). Taken together, these studies of survivin suggest that low-energy fluctuations that induce pocket formation on the protein surface are indeed a primary determinant of druggability at protein interaction sites rather than a signature of the evolved protein-binding function of these sites.

## Discussion

The results presented above support the hypothesis that the conformational transitions that open pockets at a druggable site occur with little energetic cost to the protein. This has two important consequences.

First, pocket formation within the ensemble of physiological protein fluctuations occurs only at a limited subset of surface sites. Because pocket-containing conformations are rare, it is primarily the lack of a suitable surface pocket – and not chemical composition – that renders most protein surface sites undruggable. The highly restricted druggability of protein surfaces supported by both NMR [14] and crystallographic [15] observations that probe molecules interact with a very limited subset of the protein surface. Sites at which the unbound protein crystal structure includes a pre-formed pocket (HPV E2, FKPB12, ZipA) typically exhibit only minor conformational changes that change the size or shape of this pocket, and are driven primarily by sidechain reorganization. In contrast, for the examples in which the unbound protein crystal structure does not have a pre-formed pocket or has only a small surface pocket (XIAP, MDM2, Bcl-X_L_, IL-2) concerted motions are needed that couple backbone conformational changes to the sidechain reorganization that reveals the binding pocket. In both cases, the biasing potential we describe here serves as a proxy for the binding energy associated with some (unspecified) complementary compound, and thus drives sampling towards these conformations.

Second, these results suggest that a small number of low-resolution, low energy inhibitor shapes are encoded on the protein surface through intrinsic structural and dynamic features of the protein. We propose that low-energy fluctuations produce protein conformations displaying one of these preferred shapes, at which point a complementary small-molecule ligand may be accommodated. The protein then responds to the particular steric and chemical details of the ligand via subsequent smaller conformational changes, without changing the gross features of the pocket. This model implies that exploring the pocket shapes may give clues at low-resolution to the shapes of complementary ligands, and may form the basis for a computational screening approach that matches pocket shapes to potential inhibitory ligands.

As pointed out by Cheng and colleagues [46], pocket formation is necessary but may not be sufficient for a protein surface to be druggable: the curvature and hydrophobicity of the pocket are also important. By applying their methodology to the pocket conformations generated using the biasing potential described here, it may be possible to filter for the most druggable conformations from among these pocket-containing ensembles. These conformations may not necessarily correspond to the protein-bound conformation, which has been used in some cases as a starting point for mimicry by small-molecules [47]. Generating an ensemble of druggable conformations for a given protein target may prove valuable in identifying new inhibitory compounds, even in cases where one or more inhibitors are already known. Rather than aiming to identify additional inhibitors by analogy to these known compounds, or even using the protein structure solved in complex with one of these known compounds (or the natural protein partner), matching directly against an ensemble of pocket-containing conformations removes bias towards this parent compound (or protein partner). We anticipate that the surface pockets to which the protein is most predisposed, as revealed by this approach and therefore identified without artifacts of conformational changes in response to any particular binding partner, may serve as an optimal starting point for computational screening and may facilitate identification of chemotypes unrelated to those of known inhibitors (“scaffold hopping” [3], [48]). These new scaffolds may in turn yield potent novel small molecule inhibitors through subsequent chemical elaboration.

In principle, fragment-based approaches allow multiple probe molecules to be subsequently linked together to create a single compound presenting these probe moieties in the appropriate orientation [49]. Computational solvent mapping [27], while suitable for assessing druggability in certain cases, may prove limiting for early drug discovery because of its inability to explore the concerted backbone motions necessary for thoroughly sampling the “pocket ensemble”. While FKBP sidechain motions within 6 Å of the binding site are sufficient for recognizing small probe molecules [49], the further necessity of backbone reorganization in other cases described here (XIAP, MDM2, Bcl-X_L_, IL-2) underscores the importance of backbone motions for conformational fluctuations that reveal surface pockets. Exploring these alternate conformations may further prove useful in guiding efforts to improve potency of known inhibitors, by identifying sites at which substituents can make additional strong contacts with the protein surface.

Finally, we note that the generality of our approach is conducive to its large-scale application for comparing druggability across many protein interaction sites. In the future, we expect this approach may be used to address the outstanding question of whether these few successful examples of small-molecules disrupting protein interaction sites are outliers, or whether they instead represent the first step towards an important class of new tools for therapeutic intervention.

## Methods

### Identifying pockets on protein surfaces

We implemented our modified version of the LIGSITE^cs^ [28] algorithm in the Rosetta software suite [34]. Briefly, a grid is centered at the residue of interest on the protein surface and grid points are marked as occupied by either protein (P) or solvent (S). The algorithm performs linear searches in the X, Y and Z directions as well as in each diagonal for “P-nS-P” events: cases where a line no longer than 12 Å can be drawn between two points on the protein surface that pass through only solvent, establishing this solvent region as part of a surface pocket. Subsequent additional criteria were used to eliminate spurious definition of pockets and reduce the effect of grid-based artifacts (described in **Text S1**). Adjoining grid points defined as “pocket” were clustered to determine the deep pocket volume of the largest contiguous single pocket in contact with the target residue.

### Simulation protocol

Simulations were carried out using the “relax” protocol [50] in Rosetta, which incorporates both backbone and sidechain degrees of freedom in a Monte Carlo search. To bias the simulations towards pocket-containing conformations we added to the standard energy function an additional energy term corresponding to the current deep pocket volume multiplied by a proportionality constant of −0.25 Rosetta energy units per Å^3^ (“moderate” bias). Simulations carried out with the “strong” bias used a proportionality constant of −0.25 Rosetta energy units per Å^3^. Data for each histogram was collected from 1,000 independent simulations. The simulation protocol is described in complete detail in **Text S1**.

## Supporting Information

Figure S1 **Identifying pockets on protein surfaces (complements Figure 1).** A grid is centered at the residue of interest on the protein surface (only partial grid is shown here). Grid points are classified as either “protein” (*grey background*) or “solvent” (*white background*). Linear searches (*red lines*) are used to identify and mark “protein-surface-protein” events (*green gridpoints*). These are further classified based on degree of buried (*light vs. dark green*) and adjoining grid points are clustered to yield discrete “pockets”. Only a surface pocket in contact with the surface of the “target” residue (*orange*) contributes to the biasing potential.
(EPS)

Figure S2 **Deep pocket volumes compared to Q-SiteFinder pocket volumes (complements** **Figure 1** **).** Deep pocket volumes of surface pockets at protein interaction sites harboring a bound inhibitor (*red circles*) and pockets found elsewhere on the protein surface (*black x's*) are plotted against the corresponding pocket volumes identified by Q-SiteFinder, for each of the sites used in **Figure 1B**. While the two are correlated, Q-SiteFinder volumes are typically larger than the corresponding deep pocket volumes.
(EPS)

Figure S3 **Matching of random surface sites to protein interaction sites (complements** **Figure 2** **)**. The distribution of pocket volumes identified by Q-SiteFinder for the complete set of random surface sites matched to the protein interaction sites on the basis of burial and secondary structure is shown (*black*); the corresponding pocket volumes for the protein interaction sites are also shown (*red arrows*). In order to create a matched set, only random sites with Q-SiteFinder pocket volumes greater than 100 Å^3^ were considered for **Figures 2** and **3**.
(EPS)

Figure S4 **Surface pockets emerge only at druggable sites, upon inclusion of additional random surface sites (complements** **Figure 2** **).** Pocket opening is not observed at random surface sites upon inclusion of additional sites that were previously excluded on the basis of Q-SiteFinder pocket volumes less than 100 Å^3^ (*dashed black lines*). Symbols are as defined in **Figure 2**. (**A**) Bcl-X_L_. (**B**) IL-2. (**C**) FKBP12. (**D**) HPV E2. (**E**) ZipA. (**F**) MDM2. (**G**) BIR3 domain of XIAP.
(EPS)

Figure S5 **Surface pockets emerge at druggable sites, when starting from protein-bound conformations (complements** **Figure 2** **).** Simulations were carried out only applying the biasing potential to the protein interaction site (*red*). With the exception of XIAP, results are in agreement with simulations started from the unbound protein structures (**Figure 2**). (A) Bcl-X_L_. (**B**) IL-2. (**C**) FKBP12. (**D**) HPV E2. (**E**) ZipA. (**F**) MDM2. (**G**) BIR3 domain of XIAP.
(EPS)

Figure S6 **Energetic analysis of pocket opening for the other members of our test set (complements Figure 3).** (**A**) Conformations generated using the biasing potential typically have a distribution of energies that overlaps those generated with the biasing potential, suggesting that these conformations represent low-energy states accessible to the unbound protein. (**B**) As with Bcl-xL, low-energy conformations containing large pockets are not observed for the other members of our test set unless the biasing potential is applied to the protein interaction site. Symbols are as defined in **Figure 3**.
(TIF)

Figure S7 **Representative conformations generated using the biasing potential (complements Figure 4).** An unbound crystal structure (*pink*), an inhibitor-bound crystal structure (*green, with inhibitor shown in sticks*), and a low-energy conformation generated from the unbound crystal structure using the biasing potential (*cyan, with target residue in red*) are shown for each of the proteins comprising our test set (except Bcl-X_L_, shown in Figure 4). (**A**) IL-2. (**B**) FKBP12. (**C**) HPV E2. (**D**) ZipA. (**E**) MDM2. (**F**) BIR3 domain of XIAP.
(TIF)

Figure S8 **Opening pockets shifts the protein backbone towards inhibitor-bound structures (complements** **Figure 5** **).** Distributions of backbone RMSD over interface residues to the closest inhibitor-bound crystal structure for conformations generated with (*red lines*) or without (*black lines*) the biasing potential. The backbone RMSD over interface residues from the unbound structure to the most and least similar inhibitor-bound crystal structures are also indicated (*dashed brown vertical lines*).
(EPS)

Figure S9 **Hydrophobicity of pockets generated using the biasing potential are similar to the corresponding inhibitor-bound pockets (complements Figure 5).** Distributions of percentage hydrophobic solvent accessible surface area (%hSASA) over interface atoms for conformations generated with the biasing potential (*red lines*). The lowest and highest %hSASAs from amongst all available inhibitor-bound crystal structures are indicated (*dashed green vertical lines*). (**A**) Bcl-X_L_. (**B**) IL-2. (**C**) FKBP12. (**D**) HPV E2. (**E**) ZipA. (**F**) MDM2. (**G**) BIR3 domain of XIAP.
(EPS)

Text S1 **Supplementary methods.** This supporting text contains a complete description of methodology used, including PDB structures used in calculations. A description of pocket-opened conformations for each protein in our test set is also included in this text.
(DOC)
