# Supplementary figures and images for: Druggable Protein Interaction Sites Are More Predisposed to Surface Pocket Formation than the Rest of the Protein Surface
Source: PLoS Comput Biol. 2013 Mar 7;9(3):e1002951. doi: 10.1371/journal.pcbi.1002951 (PMC3591273; doi:10.1371/journal.pcbi.1002951)

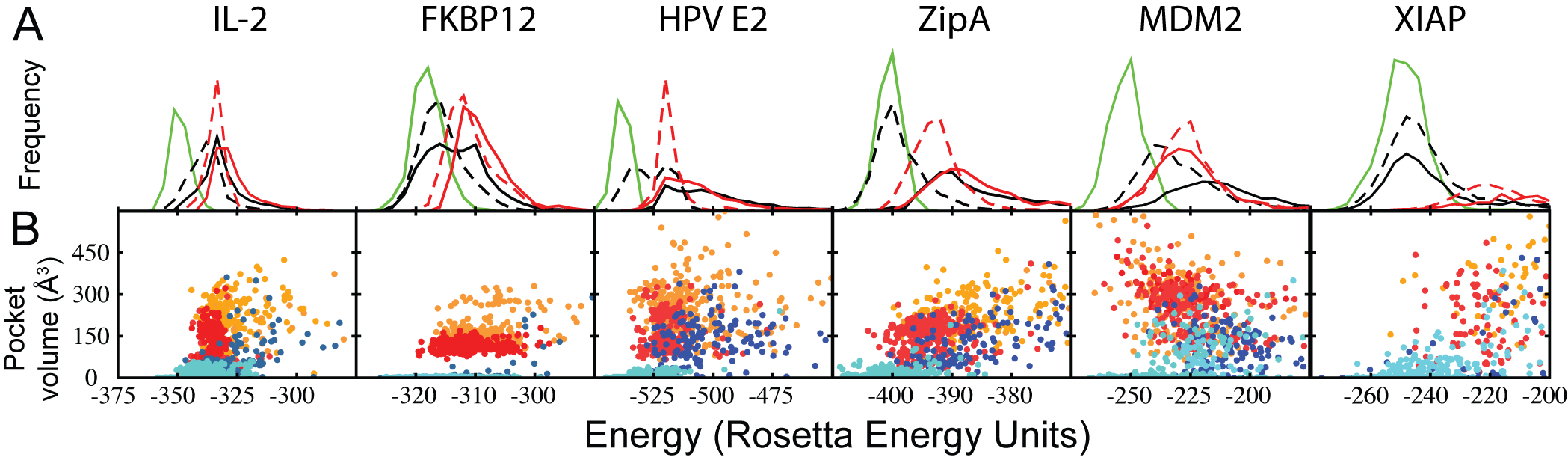

Supplement: Figure S6 — Energetic analysis of pocket opening for the other members of our test set (complements Figure 3). (A) Conformations generated using the biasing potential typically have a distribution of energies that overlaps those generated with the biasing potential, suggesting that these conformations represent low-energy states accessible to the unbound protein. (B) As with Bcl-xL, low-energy conformations containing large pockets are not observed for the other members of our test set unless the biasing potential is applied to the protein interaction site. Symbols are as defined in Figure 3 . (TIF) [file pcbi.1002951.s006.tif]

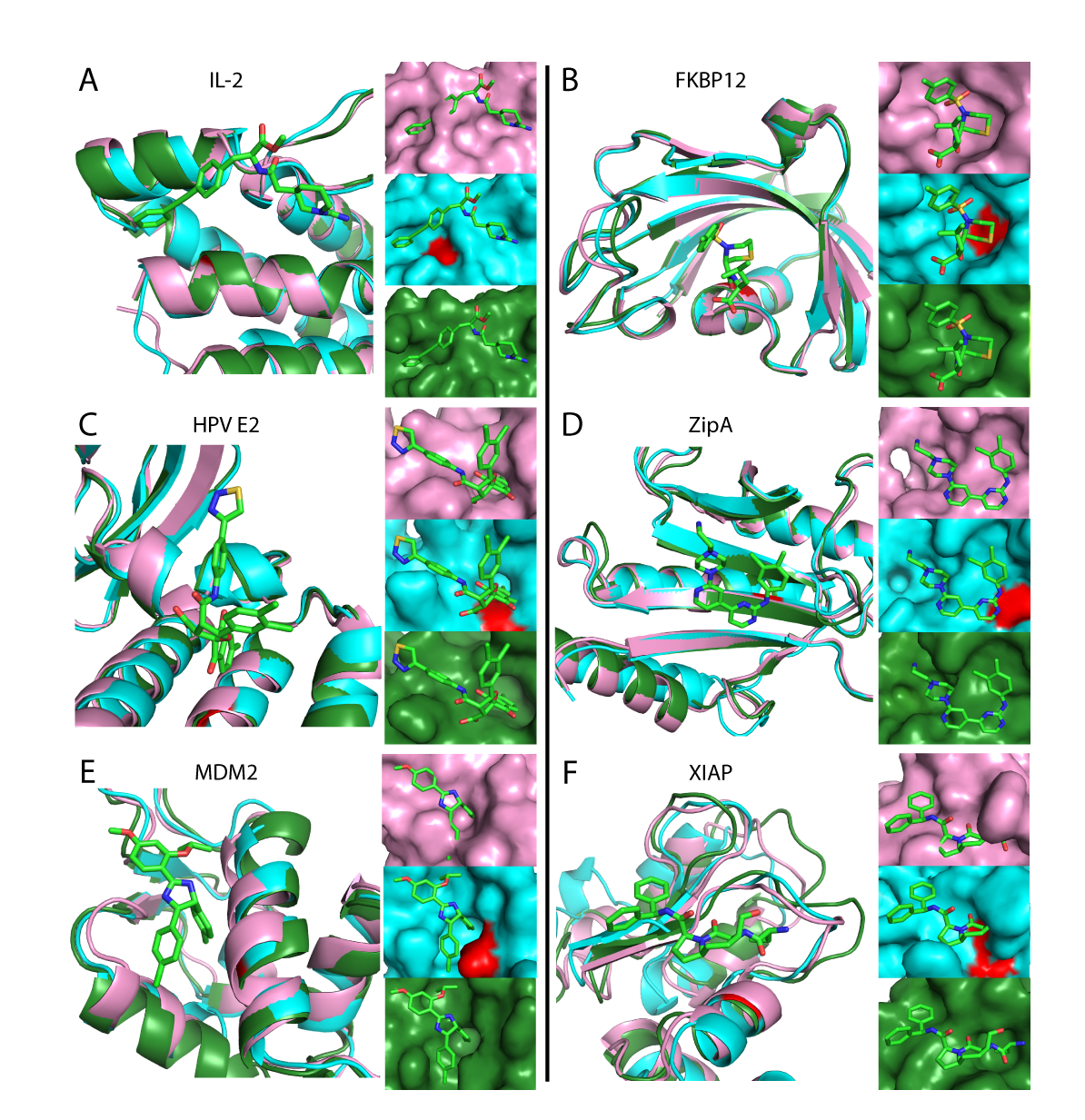

Supplement: Figure S7 — Representative conformations generated using the biasing potential (complements Figure 4). An unbound crystal structure (pink), an inhibitor-bound crystal structure (green, with inhibitor shown in sticks), and a low-energy conformation generated from the unbound crystal structure using the biasing potential (cyan, with target residue in red) are shown for each of the proteins comprising our test set (except Bcl-XL, shown in Figure 4). (A) IL-2. (B) FKBP12. (C) HPV E2. (D) ZipA. (E) MDM2. (F) BIR3 domain of XIAP. (TIF) [file pcbi.1002951.s007.tif]
