# Supplementary material for: Druggable Protein Interaction Sites Are More Predisposed to Surface Pocket Formation than the Rest of the Protein Surface
Source: PLoS Comput Biol. 2013 Mar 7;9(3):e1002951. doi: 10.1371/journal.pcbi.1002951 (PMC3591273; doi:10.1371/journal.pcbi.1002951)
Supplement: Text S1 — Supplementary methods. This supporting text contains a complete description of methodology used, including PDB structures used in calculations. A description of pocket-opened conformations for each protein in our test set is also included in this text. (DOC) [file pcbi.1002951.s010.doc]

## Text S1

### **Implementation in Rosetta**

Computational methods are implemented in the Rosetta software suite . Calculations were carried out using svn revision 48537 of the developer trunk source code. Rosetta is freely available for academic use (www.rosettacommons.org), with the new features described here included in the 3.5 release.

### **Identifying Pockets on Protein Surfaces**

A three-dimensional grid of size 20 Å x 20 Å x 20 Å and grid spacing 0.5 Å is centered at the center of mass of the “target” residue (a residue at the center of the known protein interaction site). Grid points occluded by the protein are identified using the center of mass and van der Waals radius of each heavy (non-hydrogen) atom in the protein. The protein surface is then mapped using an expanded radius that includes a 1.0 Å buffer region, and surface points in direct contact with the target residue are marked as such. Grid points not occluded by the protein (including “surface” points) are labeled “solvent”.

For each “surface” point on the grid, a ray is traced in each direction of the six principle axes and four diagonals (corresponding to half the faces and half the corners of a cube centered at this grid point). Solvent points are marked as “pocket” when a ray contains a surface point followed by at least 3 Å, but no more than 12 Å, of consecutive solvent points followed by another surface point (**Figure S1**).

Though appropriately identifying known inhibitor binding sites, this algorithm proved susceptible to defining certain classes of spurious pockets not suitable for interacting with small-molecules. The most common were surface (saddle-shaped) “channels,” in which the pocket is bounded on only two sides. To eliminate these, our algorithm carries out a second search along the same 10 directions. Any grid points marked as “pocket” that are bounded on both sides by only solvent are reverted to solvent themselves (**Figure S1**).

Adjacent grid points defined as “pocket” are used to determine the volume of the largest contiguous single pocket in contact with the “target” residue. The pocket volume is taken to be the number of pocket points that are not within 2.5 Å of a solvent grid point, multiplied by 0.125 Å^3^ (the volume associated with each point based on the 0.5 Å grid spacing). To eliminate effects of the precise grid orientation with respect to the protein, the grid is randomly rotated and the pocket identification is repeated. The final pocket volume is taken to be the pocket volume averaged over 100 rotations of the grid.

The Rosetta command line used to determine the pocket volume as described above is as follows (for target residue number 108):

pocket_measure.linuxgccrelease ‑s input_pdb ‑num_angles 100 ‑pocket_max_spacing 12 ‑pocket_psp false ‑pocket_sps ‑central_relax_pdb_num 108

### **Selection of random surface residues**

Random surfaces were selected using the following criterion: secondary structure in the unbound structure matches those of the target residues, the residue must be at least 12 Å from the protein interaction site, and the residue must be at least 12 Å from all other residues selected so far. The selection process was carried out iteratively until no additional residues met these criteria. Only those with pocket size (evaluated by Q-SiteFinder ) matching the protein interaction site (**Figure S3**) were included in **Figures 2** and **3**).

### **Simulation protocol**

Simulations were carried out using the now-default “fast-relax” variation of the relax protocol in Rosetta, with an additional biasing term proportional to the volume of the target pocket (in Å^3^) included in the standard Rosetta energy function. The energetic contribution of this term was capped when the pocket volume reached 300 Å^3^. In order to reduce simulation time, we computed pocket volume using only two random grid orientations (as opposed to averaging over 100 rotations of the grid as above). The fast-relax protocol used in these studies was comprised of five cycles (Monte Carlo steps) per trajectory, where each cycle entailed repacking of the side chains with the repulsive part of the van der Waals term incrementally ramped up in weight, followed by a gradient-based energy minimization. The last of these, the gradient-based energy minimization, formally requires derivatives of the pocket volume with respect to the protein atomic coordinates that are not solvable analytically. For this reason we set the derivatives of the “pocket” contribution to zero, meaning that this minimization step is carried out without regard to the pocket size. Despite this, application of the Metropolis criterion using energies that include this “pocket” contribution nonetheless serves to bias the resulting trajectory towards pocket-containing conformations.

The Rosetta command line to carry out the unbiased simulation described above is as follows:

relax.linuxgccrelease ‑relax:fast ‑s input.pdb ‑nstruct 1000

The equivalent biased simulations were carried out by adding the command-line flags:

‑pocket_zero_derivatives ‑pocket_max_spacing 12 ‑pocket_psp false ‑pocket_sps ‑pocket_num_angles 2 ‑score:weights score12 ‑score:patch pocket.wts.patch ‑cst_fa_file constraints.txt

The pocket.wts.patch file contains the single line “pocket_constraint = 1.0” and the constraints.txt file contains a single line of the form “pocket_weight target_residue” where the pocket weight has units REU per Å^3^ and target_residue can have formats such as 108 or A:108.

### **Unbiased energetic evaluation of conformations**

The pocket-opened structures were minimized with Rosetta using the standard Rosetta energy function to remove any clashes, and the final (unbiased) energy was reported again using the standard energy function.

This minimization step was carried out using the Rosetta command line:

minimize.linuxgccrelease ‑in:file:fullatom ‑s input.pdb

### **iRMSD calculations**

Interface residues were defined as residues that have either an attractive or repulsive van der Waals (as implemented in Rosetta) interaction with the inhibitor of absolute magnitude greater than 0.01 REUs. All protein Cα atoms were used for alignment, then iRMSD was computed over all non-hydrogen atoms of the interface residues.

### **PDB structures used in calculations**

Results were described for seven proteins comprising our test set: Bcl-X_L_, IL-2, FKBP12, HPV E2, ZipA, MDM2, and the BIR3 domain of XIAP.

All unbound calculations (and starting points for simulations) used structures with the following PDB IDs: 1R2D (Bcl-X_L_), 1M47 (IL-2), 2PPN (FKBP12), 1R6K (HPV E2), 1F46 (ZipA), 1Z1M (MDM2), and 1F9X (BIR3 domain of XIAP). For NMR structures (1Z1M and 1F9X), we used the first model in the NMR ensemble as a starting point.

The pocket volumes shown in Figure 1B were calculated from bound structures with the following PDB IDs: 2YXJ (Bcl-X_L_), 1M48 (IL-2), 1BKF (FKBP12), 1R6N (HPV E2), 1S1J (ZipA), 1RV1 (MDM2), 2JK7 (BIR3 domain of XIAP).

The iRMSD calculations to the bound conformations in Figure 5 used the following bound structures with PDB IDs. Bcl-X_L_: 1YSG, 1YSI, 1YSN, 2O22, 2O2M, 2O2N, and 2YXJ. IL-2: 1M48, 1M49, 1M4A, 1M4B, 1PW6, 1PY2, and 1QVN. FKBP12: 1A7X, 1BKF, 1BL4, 1F40, 1FKB, 1FKD, 1FKF, 1FKG, 1FKH, 1FKI, 1FKJ, 1J4H, 1J4I, 1J4R, 1QPF, 1QPL, 2DG3, 2DG4, 2DG9, and 2FKE. HPV E2: 1R6N. ZipA: 1S1J, 1S1S, 1Y2F, and 1Y2G. MDM2: 1RV1, 1T4E, and 1TTV. BIR3 domain of XIAP: 1TFQ, 1TFT, 2JK7, 2OPY, 3CLX, 3CM2, 3CM7, and 3EYL.

Calculations described in Figure 6 were started from the unbound survivin structure, PDB ID 1E31.

### **Description of pocket-opened conformations**

The pocket-opening of Bcl-X_L_ is described in the main text.

IL-2 (**Figure S7A)** is similar to Bcl-xL in that a large clash is found when superimposing the ligand onto the unbound structure. Several of the helical regions align well, but there are differences in the alignment of the helix located behind the left side of the small molecule. This allows a side chain to move and resolve the clash while at the same time allowing narrowing the left side of the pocket, allowing more interaction with the inhibitor.

FKBP12 (**Figure S7B**), HPV E2 **Figure S7C**), and ZipA (**Figure S7D**) are each cases where the pockets are largely pre-formed. The backbones of the bound, unbound, and pocket-opened structure of FKBP12 all are in very close alignment with the exception of some movement of a loop region to make a more favorable interaction with the ligand in the bound structure. This movement is partially recapitulated in the opened structure. Likewise, superimposing the small molecule onto the unbound structure yields no clashes. This is consistent with the distribution of pocket sizes (**Figure 2C**) where two of the target residues had only a small increase in the pocket size when the biasing potential was applied. HPV E2 is also largely pre-formed, though there is a clash at the deepest part of the small molecule pocket. The backbones differ little, though the clash is resolved in the bound structure by the slight translation of the helix containing the target residue and the rotation of a histidine side chain. ZipA is also lacking in clashes and the backbone largely aligns around the interface. When larger pockets were identified through biased simulations, the expansion was largely confined to the area adjacent to the binding site on the left side of the figures.

MDM2 (**Figure S7E**) has several significant clashes when the small molecule is superimposed onto the binding site. There are essentially two motions going on that open the pocket. The helix to the right wobbles to the right relieving two clashes with the small molecule while the helix below the small molecule tilts away, relieving the other clash. These motions were recapitulated in the biased simulation and yield visually impressive results.

The interface of the BIR3 domain of XIAP (**Figure S7F**) is somewhat disordered and has a large clash on one side when the small molecule is superimposed onto the unbound structure, and a pre-formed pocket on the other side. The pocket is opened by a loop moving out of the way, along with some compensatory wobble motion with a helix that the loop attaches to.

## Supplementary References

1. Leaver-Fay A, Tyka M, Lewis SM, Lange OF, Thompson J, et al. (2011) ROSETTA3: an object-oriented software suite for the simulation and design of macromolecules. Methods Enzymol 487: 545-574.

2. Laurie AT, Jackson RM (2005) Q-SiteFinder: an energy-based method for the prediction of protein-ligand binding sites. Bioinformatics 21: 1908-1916.

3. Qian B, Raman S, Das R, Bradley P, McCoy AJ, et al. (2007) High-resolution structure prediction and the crystallographic phase problem. Nature 450: 259-264.
